# The proper use of coronary calcium score and coronary computed tomography angiography for screening asymptomatic patients with cardiovascular risk factors

**DOI:** 10.1038/s41598-017-17655-w

**Published:** 2017-12-15

**Authors:** Shee Yen Tay, Po-Yen Chang, Wilson T. Lao, Ying Chin Lin, Yi-Han Chung, Wing P. Chan

**Affiliations:** 1 Department of Radiology, Wan Fang Hospital, Taipei Medical University, Taipei, 116 Taiwan, Republic of China; 2 Department of Radiology, School of Medicine, College of Medicine, Taipei Medical University, Taipei, 110 Taiwan, Republic of China; 3 Department of Family Medicine, Shuang Ho Hospital, Taipei Medical University, New Taipei City, 235 Taiwan, Republic of China; 4 Department of Family Medicine, School of Medicine, College of Medicine, Taipei Medical University, Taipei, 110 Taiwan, Republic of China

## Abstract

Early detection and treatment of coronary artery disease (CAD) can reduce incidences of acute myocardial infarction. In this study, we determined the proper use of contributing risk factors and coronary artery calcium score (CACS) when screening asymptomatic patients with coronary arterial stenoses using coronary computed tomography angiography (CCTA). We reviewed 934 consecutive patients who received CACS and CCTA between December 2013 and November 2016. At least one cardiovascular disease risk factor was present in each of the 509 asymptomatic participants. Patients were grouped based on CACS into “zero,” “minimal” (0 < CACS ≤ 10), “mild” (10 < CACS ≤ 100), “moderate” (100 < CACS ≤ 400), and “excessive” (CACS > 400). Males over 45 years old with diabetes mellitus and hypertension had a higher risk of significant coronary stenosis. In multivariate analysis, age, sex, hypertension, and diabetes mellitus remained significant predictors of stenosis. A CACS of zero occurred in 227 patients (44.6%). There were no significant differences between the “zero” and “minimal” groups (*p = *0.421), but the “mild,” “moderate,” and “excessive” groups showed correlations with significant coronary stenosis. Age, sex, diabetes mellitus, and hypertension were associated with higher risk of significant coronary stenosis. Asymptomatic patients with CACSs of zero do not require CCTA, and thereby avoid unnecessary radiation exposure.

## Introduction

Coronary artery disease (CAD) is a major cause of death in developed countries. Although CAD mortality rates have declined globally over the past four decades, it remains responsible for approximately one-third of all deaths of individuals aged over 35 years old^1,2^. According to estimates, nearly one-half of men and one-third of women over 40 years old will develop some symptoms of CAD in the United States^3^. In Taiwan, coronary heart disease has been the second leading cause of death for both men and women in the past 10 years, and the mortality rate of CAD increased 8.1% in 2016 (88.5/100,000) compared to 2015 (81.8/100,000)^4^.

Many CAD patients are asymptomatic, and early detection and treatment of CAD can reduce the incidence of acute myocardial infarction^5,6^. Identifying the coronary arterial plaque pattern can provide more effective treatment methods. Non-calcified plaque has been shown to have a higher tendency to regress in response to established medical therapies^7^. Recent developments in medical technologies, such as dual-source computed tomography (DSCT), have facilitated scanning the heart and coronary arteries noninvasively with high image quality and low radiation dose^8–10^. Multiple studies have shown that coronary artery stenoses can be identified with high sensitivity, specificity, positive predictive value, and negative predictive value via coronary computed tomography angiography (CCTA), given a sufficiently high image quality^9,11^.

Studies have also shown that the coronary artery calcium score (CACS) is useful for refined risk stratification, particularly with high-risk patients and those with diabetes mellitus (DM)^12,13^. Asymptomatic patients at high-risk will likely benefit from increased medical surveillance^5^. However, asymptomatic risk-free patients should not be screened^14^. The aim of this study is to determine the proper use of contributing risk factors and CACS in screening patients with coronary arterial stenoses using CCTA.

## Results

In total, 509 asymptomatic patients were recruited for this study. The mean (±SD) ages were 57.79 (±10.60) years for the 344 males and 58.84 (±10.00) years for the 165 females studied. Males over 45 years old were at higher risk of significant stenosis than those under 45 (OR: 3.541, p = 0.041). There was also a higher risk among men with hypertension (OR: 2.862, p < 0.001) and DM (OR: 3.803, p < 0.001) (Table 1), which was also the case for females (age > 55 years old, OR: 142.5, p = 0.01; hypertension: OR: 5.479, p = 0.031; DM: OR: 5.357, p < 0.01; Table 2). Patients with at least three risk factors had a higher prevalence of significant coronary stenosis (OR: 6.078, p < 0.001; Table 3). The seven patients who had significant coronary stenosis with fewer than three risk factors had non-zero CACS. There were 91 out of 149 patients with zero CACSs and fewer than three risk factors who had no stenosis or stenosis less than 50% (Table 3).

**Table 1:** Difference in the prevalence of significant coronary stenosis detected by coronary computed tomography angiography with risk factors of coronary artery stenosis compared to those without them in male patients.

Risk factor	Total number of patients (%)	Number of patients with significant coronary stenosis	*p*-value	OR (95% CI)
**Age**				
M > 45	308 (89.5)	75	0.041*	3.541 (1.056–11.876)
M ≦ 45	36 (10.5)	3		
**BMI**				
≧27	105 (30.5)	21	0.433	0.798 (0.455–1.402)
<27	239 (69.5)	57		
**Hypertension**				
Yes	215 (62.5)	62	<0.001***	2.862 (1.569–5.220)
No	129 (37.5)	16		
**Dyslipidemia**				
Yes	185 (53.8)	43	0.786	1.073 (0.646–1.781)
No	159 (46.2)	35		
**Diabetes**				
Yes	76 (22.1)	33	<0.001***	3.803 (2.183–6.627)
No	268 (77.9)	45		
**Smoke**				
Yes	86 (25.0)	18	0.656	0.874 (0.482–1.583)
No	258 (75.0)	60		
**Lack of exercise**				
Yes	180 (52.3)	45	0.281	1.323 (0.795–2.202)
No	164 (47.7)	33		
**AMI** ^**+**^ **family history**				
Yes	73 (21.2)	11	0.084	0.540 (0.269–1.086)
No	271 (78.8)	67		

*P < 0.05, **P < 0.01,***P < 0.001.

^+^Acute myocardial infarction.

**Table 2:** Difference in the prevalence of significant coronary stenosis detected by coronary computed tomography angiography with risk factors of coronary artery stenosis compared to those without them in female patients.

Risk factor	Total number of patients (%)	Number of patients with significant coronary stenosis	*p*-value	OR (95% CI)
**Age**				
>55	108 (65.5)	12	0.01	142.5 (0.0217–935579.559)
≦55	57 (34.5)	0		
**BMI**				
≧27	31 (18.8)	2	0.845	0.855 (0.178–4.115)
<27	134 (81.2)	10		
**Hypertension**				
Yes	83 (50.3)	10	0.031*	5.479 (1.162–25.841)
No	82 (49.7)	2		
**Dyslipidemia**				
Yes	95 (57.6)	9	0.216	2.337 (0.609–8.972)
No	70 (42.4)	3		
**Diabetes**				
Yes	23 (13.9)	5	<0.01**	5.357 (1.537–18.672)
No	142 (86.1)	7		
**Smoke**				
Yes	4 (2.4)	1	0.206	4.545 (0.436–47.401)
No	161 (97.6)	11		
**Lack of exercise**				
Yes	93 (56.4)	7	0.886	1.091 (0.331–3.590)
No	72 (43.6)	5		
**AMI family history**				
Yes	29 (17.6)	2	0.932	0.933 (0.193–4.504)
No	136 (82.4)	10		

*P < 0.05, **P < 0.01,***P < 0.001.

**Table 3:** Prevalence of significant coronary stenosis detected by coronary computed tomography angiography with the number of risk factors including age (males > 45 yrs, females > 55 yrs), obesity (BMI > 27), hypertension, dyslipidemia, diabetes, smoking, lack of exercise, AMI family history.

Risk factor number	Total number of patients (%)	Number of patients with significant coronary stenosis	*p*-value	OR (95% CI)
<3	149 (29.3)	7		
≧3	360 (70.7)	83	< 0.001***	6.078 (2.738–13.494)

*P < 0.05, **P < 0.01,***P < 0.001.

**Figure 1:**
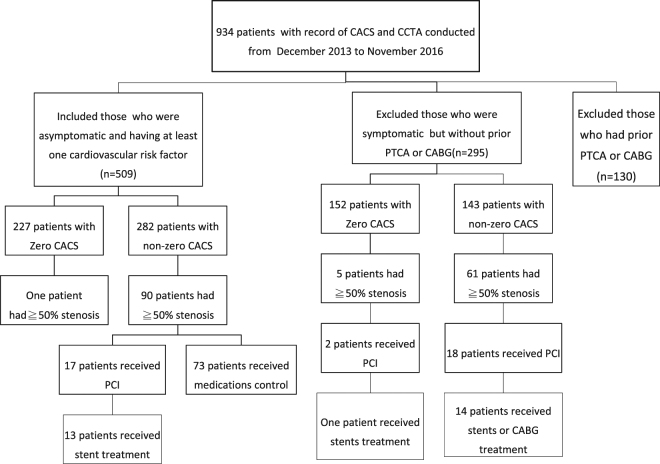
Flow chart of the study population. Abbreviations: CACS: coronary arterial calcium score; CCTA: coronary CT angiography; PTCA: percutaneous transluminal coronary angioplasty; CABG: coronary artery bypass grafting; PCI: percutaneous coronary intervention.

According to the multivariate analysis for risk factors of CAD, sex (OR: 3.089, p < 0.01), age (OR: 5.025, p = 0.01), hypertension (OR: 2.972, p < 0.001), and DM (OR: 3.901, p < 0.001) remained major predictors of significant coronary stenosis in at least one segment of the coronary arteries in CCTA (Table 4). In the CCTA, 227 patients were found to have a CACS of zero (44.6%; Table 5). One male (0.44%) out of the 227 had significant coronary stenosis in at least one segment of his coronary arteries (Fig. 1). This patient had three risk factors (hypertension, hyperlipidemia, and age > 45 years). No notable difference was observed in significant coronary stenosis among the patients with a CACS of 0–10 (OR: 3.139, p = 0.421). However, CACSs of 10–100 (OR: 43.566, p < 0.001), 100–400 (OR: 284.323, p < 0.001), and > 400 (OR: 1065.429, p < 0.001) showed noteworthy correlations with significant coronary stenosis (Table 5).

**Table 4:** Multivariate analysis of significant coronary artery stenosis detected by coronary computed tomography angiography with risk factors.

	*p*-value	OR (95% CI)
Sex	<0.01**	3.089 (1.526–6.251)
Age	0.0099*	5.025 (1.471–17.164)
BMI ≧ 27	0.057	0.564 (0.313–1.016)
Hypertension	0.001***	2.972 (1.650–5.352)
Dyslipidemia	0.768	0.925 (0.550–1.554)
DM	0.001***	3.901 (2.247–6.771)
Smoking	0.947	0.979 (0.522–1.837)
Lack of exercise	0.120	1.501 (0.899–2.507)
AMI family history	0.076	0.539 (0.273–1.067)

Age: Men > 45 yrs; women > 55 yrs.

*p < 0.05, **p < 0.01, ***p < 0.001.

**Table 5:** Distribution of different coronary artery calcium score groups in relation to the presence of nonobstructive or obstructive coronary artery disease.

Calcium score	Total number of patients (%)	Number of patients with significant coronary stenosis	*p*-value	OR (95% CI)
Zero (score = 0)	227 (44.6)	1		
Minimal (0 < score ≦ 10)	73 (14.3)	1	0.421	3.139 (0.194–50.822)
Mild (10 < score ≦ 100)	99 (19.4)	16	<0.001***	43.566 (5.688–333.675)
Moderate (100 < score ≦ 400)	70 (13.8)	39	<0.001***	284.323 (37.713–2143.567)
Excessive (score > 400)	40 (7.9)	33	<0.001***	1065.429 (127.008–8937.565)

*p < 0.05, **p < 0.01, ***p < 0.001.

Significant coronary stenosis was found in 90 out of 282 (31.91%) participants with non-zero CACSs (Fig. 1). We referred these 90 patients to cardiologists, and 17 of them underwent percutaneous coronary intervention (PCI) and 13 patients (14.4%) received stent treatments. The mean (±SD) radiation dose for calcium scans for all patients was 0.50 (±0.18) mSv, and for CCTA was 2.66 (±1.61) mSv (BMI < 24, n = 216) and 4.92 ± 3.78 mSv (BMI > 27, n = 130). Finally, the mean score for image quality was 2.99 (±0.65).

## Discussion

In our study, DM and hypertension increased the risk of coronary stenosis in both male and female patients with CAD detected via CCTA. This shows that individuals with DM have a higher prevalence of CAD and are more likely to suffer a myocardial infarction and/or silent myocardial ischemia^15^. In the Framingham Heart Study, DM doubled and tripled the age-adjusted risk for cardiovascular disease in males and females respectively^16^. The Emerging Risk Factors Collaboration Group performed a meta-analysis of 102 studies that included 530,083 patients with no history of myocardial infarction, angina, or stroke. After adjusting for other risk factors, patients with DM had twice the overall risk of CAD, as well as a higher risk of cardiac death and non-fatal myocardial infarction^17^. In patients with type 2 DM, the risk of diabetic complications was strongly correlated with high blood pressure.

In our own multivariable analysis, risk factors that confer increased risk of CAD include being male and having older age, hypertension, and diabetes. In a Korean study, age and sex were the strongest overall predictors of atherosclerosis as assessed by CCTA in an asymptomatic population of 1,015 subjects. However, these two risk factors were not particularly associated with a specific sub-type of coronary plaque^18^. According to the diagnosis, hypertension was present in many patients with type 2 DM^19^. The lifetime risk of developing CAD is significantly higher among patients with hypertension. In a cohort of over 1.25 million patients aged 30 years or older without baseline CAD (including 20% with treated hypertension at baseline), patients with baseline hypertension had a 63.3% lifetime risk of developing CAD compared to 46.1% for those with non-elevated baseline blood pressure^20^.

Atherosclerosis begins with the development of fatty streaks (focal thickening of the intima with accumulation of lipid-laden macrophages and extracellular matrix)^21^. These streaks become fibrous plaques via the accumulation of connective tissue and an increased number of smooth muscle cells filled with lipids. More advanced lesions contain a necrotic lipid-rich core and eventually calcified regions^22^. Because the advanced lesions of atherosclerosis occur with increasing frequency in older populations^22^, the appearance of a calcified plaque indicates the possible presence of soft and vulnerable plaques, which can only be observed using CCTA.

We propose that CACS could be measured at a low radiation dose, which would cause less harm to low-risk patients. CCTA may not be required if the CACS is zero. According to guidelines from the European Society of Cardiology and the American College of Cardiology/American Heart Association (ACC/AHA), CACS is considered an acceptable option (Class IIa) for primary screening of asymptomatic patients with Framingham intermediate risk (10–20% 10-year risk)^23,24^. The guidelines also note that the measurement of arterial calcium is reasonable for patients at low to intermediate risk (6–10% 10-year risk). Arterial calcium measurement is not recommended for patients at low (<6% 10-year risk) or high risk.

In our study, only one patient had three risk factors with a CACS of zero; however, he had significant coronary stenosis in at least one segment of his coronary arteries. This patient was referred to the cardiology outpatient department for a thallium scan, which showed only mild stress-induced ischemia that was reversible with rest. Overall, CACS is highly predictive of the absence of significant coronary artery stenosis. Although a CACS of 0–10 wasn’t found to significantly correlate with coronary stenosis, this requires further study, as our patient population in this range was not sufficiently large.

A series of studies revealed that patients with CACSs of zero had <1% probability of significant coronary stenosis [reviewed in^25^]. Many researchers have found that asymptomatic patients with at least one risk factor for CAD and a CACS of zero have a good prognosis^14,26,27^. According to Dedic *et al*.^14^, the annual incidence of adverse events (all-cause mortality, nonfatal myocardial infarction, and unstable angina) in patients with CACSs of zero (0.4% [CI 0.1% to 1.3%]) was significantly lower compared to patients with CACSs over 400 (6.8% [CI 4.8% to 9.4%]). Thus, such patients may not require further imaging with CCTA. However, Plank *et al*. revealed that a CACS of zero could not exclude CAD, so patients should undergo CCTA if they have diabetes or low lifetime risk, with borderline or non-specific mild pathology in the ECG-treadmill stress test^28^.

However, a CACS of zero does not carry the same highly negative predictive value in symptomatic patients. In the CONFIRM registry, which involved 10,037 patients who underwent both CACS and CCTA, 13% of patients with a CACS of zero had non-obstructive stenosis and 3.5% had significant stenosis^29^. Therefore, in symptomatic patients with a CACS of zero, obstructive CAD is possible and associated with increased cardiovascular events. Taken together, CACS does not add additional prognostic information to CCTA in symptomatic patients. In our study, six of 152 (3.9%) symptomatic patients with a CACS of zero had significant stenosis in at least one coronary artery (Fig. 1).

Furthermore, 90 out of 282 (31.9%) non-zero CACS patients had significant coronary stenosis, but only 17 of that 90 (18.9%) underwent PCI. Because most participants had moderate stenosis (50–69% stenosis), pharmaceutical intervention was preferred over performing PCI. CCTA is required for noncalcified plaque evaluation in patients with a positive finding from calcium scanning. Arterial calcium scanning may not identify unstable plaques that are prone to rupture directly, but areas of arterial calcium tend to colocalize with vulnerable and/or unstable plaques, and patients with high CACS are likely to have a large burden of noncalcified plaques^30^. Thus, patients with high CACS have a higher likelihood of plaques that are prone to rupture and should undergo CCTA.

In a study of 422 individuals, the rate of conversion to a positive CACS from zero was 25% over a mean period of 4.1 ± 0.9 years, and was associated with age, DM, and cigarette smoking^31^. The rate was nonlinear, and the highest rate of conversion occurred in the fifth year. No clinical factor appeared to mandate earlier repeat CACS before four years.

Examinations performed to determine CACS generally involve a lower radiation dose than required for CCTA. In our study, the mean radiation dose for CACS was 0.50 mSv. A low dose of CACS is harmless and suitable for screening. Although the presence and extent of arterial calcium is predictive of coronary artery stenosis in general, it is a better indicator of the extent of coronary atherosclerosis than of stenosis severity.

The predictive value is markedly lower than generally reported for CCTA in the detection of significant coronary artery stenosis in patients with low to intermediate CAD risk who are clinically referred for non-invasive evaluation of chest pain^32^. According to Arbab-Zadeh *et al*., CCTA is less effective in patients with a CACS ≥ 600 and in patients with a high pre-test probability for obstructive CAD^33^.

A few limitations were inherent to this study. First, we did not follow up every participant without significant coronary stenosis, and the incidences of adverse events are not fully known. Second, our study population was not large enough to determine whether patients with a CACS of zero and three risk factors should undergo CCTA for noncalcified plaque evaluation. Third, our study did not consider smoking status (non-smokers, current smokers, former smokers)^34^, and therefore the relationship between smoking and CAD risk could not be evaluated.

In conclusion, CACS and CCTA represent reliable, non-invasive tools for the identification of coronary artery stenosis in asymptomatic patients. Being male, aged, and having hypertension and/or diabetes are the four most impactful risk factors for the development of significant coronary artery stenosis. Furthermore, asymptomatic patients with CACSs of zero and fewer than three risk factors may not require CCTA, thereby avoiding unnecessary radiation exposure.

## Methods

### Study population

This study was reviewed and approved by the Joint Institutional Review Board of Taipei Medical University (TMU-JIRB-N201706022). The informed consent requirement was waived due to the study’s retrospective nature. All methods were performed in accordance with relevant guidelines and regulations.

The imaging protocol and clinical data were collected in a structured case log, with one clinical nurse assigned to every patient. We retrospectively reviewed patients who underwent CACS and CCTA at our institution between December 2013 and November 2016. Criteria for inclusion were being asymptomatic and having at least one cardiovascular risk factor such as hypertension, dyslipidemia, DM, smoking, lack of exercise, and family history of myocardial infarction in first-degree relatives. Exclusion criteria included having incomplete charts, being symptomatic, having a history of coronary heart disease (myocardial infarction or angina), and having had a coronary artery bypass graft or percutaneous transluminal coronary angioplasty. After applying the criteria, 509 patients were included (Fig. 1).

### Cardiovascular risk factors

Participants’ data were collected via questionnaires and laboratory tests. Subjects were considered hypertensive if they were prescribed anti-hypertension medicine, had been clinically diagnosed with hypertension, or had either a systolic blood pressure (SBP) ≥ 140 mmHg or a diastolic blood pressure (DBP) ≥ 90 mmHg. Patients who met one of the following requirements were identified as having DM: taking an oral hyperglycemic agent, using insulin, having a clinical diagnosis of DM, or having a serum fasting glucose level > 126 mg/dL (7.0 mmol/l) or >200 mg/dl (11.1 mmol/l) two hours after an oral glucose tolerance test. They were identified as having dyslipidemia if they met one of the following requirements: diagnosis of hypercholesterolemia, medication history for hypercholesterolemia, total cholesterol >200 mg/dL (5.172 mmol/l), or low-density lipoprotein >130 mg/dL (3.367 mmol/l). Approximately 98% of patients had two measurements recorded for blood pressure, blood glucose, and lipid profile.

Body mass index (BMI) was calculated by dividing participants’ body weight (in kilograms) by their height (in meters) squared. In Taiwan, obesity is defined as BMI ≥ 27 kg/m^2^. Subjects were identified as current smokers if they had smoked within the year leading up to the survey date and as former smokers if they had quit at least one year before the survey date. Subjects who did not exercise regularly at least three times a week were categorized as lacking exercise.

### Scan protocol and image reconstruction

Patients with a heart rate over 70 bpm received 20–60 mg of propranolol orally based on body mass every 30 minutes until the rate dropped below that level. All patients prescribed propranolol were first cleared by a clinician for contraindications. Nitroglycerin was not administered to any of the participants.

Patients’ heart rates and electrocardiograms (ECG) were monitored during the scan. An ECG-triggered CACS was performed first. All CACSs were calculated using the Agaston score, with patients being classified into five groups: “zero” (CACS = 0), “minimal” (0 < CACS ≤ 10), “mild” (10 < CACS ≤ 100), “moderate” (100 < CACS ≤ 400), and “excessive” (CACS > 400)^35,36^. All CT scans were performed using a second-generation dual-energy CT system (SOMATOM Definition Flash, Siemens Healthcare, Forchheim, Germany) with automated features for tube current and voltage selection (Siemens Care Dose 4D and Siemens CARE kV; detector collimation 128 × 0.6 mm; kVp 100 or 120; gantry rotation time 0.28 sec; slice thickness 0.7 mm).

The dosage of contrast medium was tailored to ensure aortic enhancement using the test-bolus technique provided by the manufacturers. The test boluses were conducted with 15 ml of contrast media, followed by 20 ml of saline. The following scanning parameters were employed: an 80 ml bolus of contrast medium (Ultravist 370 mg/ml, Berlex) was injected with an 18 gauge intravenous catheter placed in the antecubital vein using a dual-head power injector (Mallinckrodt, Santa Monica, CA, USA). The default injection rate was 5 ml/sec, which was reduced to 4 ml/sec if the patient could not tolerate the initial rate. The volumetric quantity of administered contrast medium was determined using the following equation:

$${\rm{Total}}\,{\rm{volume}}\,{\rm{of}}\,{\rm{injection}}[{\rm{ml}}]={\rm{v}}[\text{ml}/{\rm{s}}]\times (7\,{{\rm{T}}}_{{\rm{peak}}})[{\rm{s}}]$$

where v is the injection rate of 5 ml/s, and T_peak_ represents the time to peak contrast enhancement in seconds.

Patients were instructed to breathe quietly before the administration of the contrast and to hold their breath during the actual scanning. To estimate the effective dose of cardiac scans, the CT dose index volume (CTDIvol) was multiplied by the scan length and a standard thoracic conversion coefficient of 0.014 mSv (mGy cm)^−1^. Three-dimensional images were reconstructed and viewed on a dedicated workstation (3D Workplace, Siemens, Erlangen, Germany) using cardiac post-processing software (SyngoVia CT Coronary, Siemens, Erlangen, Germany). To ensure optimal assessment of each coronary artery, the reconstructed images were selected from variable phases of the cardiac cycle by one of five radiologists.

### Image evaluation

Coronary stenosis data were gathered from the case log record. All CCTA images were interpreted by five radiologists who specialized in this area. Next, a previously described 17-segment American Heart Association model of the coronary tree was implemented^37^. In human and animal studies, a stenosis that reduces the lumen diameter by 50% can result in a three- to four-fold reduction in coronary bed flow reserve^38,39^. An obstructed coronary vessel was defined as a ≥50% reduction in the diameter of the lumen^40^. The image quality for each patient was assessed on a 4-point scale (4: excellent; 3: good; 2: average; 1: poor)^41^. For each participant, the lowest score found in any coronary segment was recorded.

### Statistical analysis

SPSS version 18 (SPSS Inc.; Chicago, IL, USA) was used for statistical analysis. The means and standard deviations were used to depict continuous variable distributions, while frequency counts were used to summarize categorical variable distributions. The binary logistic regression was used to estimate the probability of a binary response based on one or more independent variables. Multiple logistics regression analysis was used to reveal associations between sex, age, BMI ≧ 27 hypertension, dyslipidemia, DM, smoking, lack of exercise, AMI family history, and coronary artery stenosis status. A p-value < 0.05 was considered statistically significant.

## References

[1] Lloyd-Jones D (2010). Executive summary: heart disease and stroke statistics–2010 update: a report from the American Heart Association. Circulation.

[2] Nichols M, Townsend N, Scarborough P, Rayner M (2014). Cardiovascular disease in Europe 2014: epidemiological update. European heart journal.

[3] Lloyd-Jones DM, Larson MG, Beiser A, Levy D (1999). Lifetime risk of developing coronary heart disease. Lancet (London, England).

[4] Welfare, M. o. H. a. *Ten leading causes of dealth in Taiwan*, http://www.mohw.gov.tw/cp-16-33598-1.html (2017).

[5] Robinson JG, Gidding SS (2014). Curing atherosclerosis should be the next major cardiovascular prevention goal. Journal of the American College of Cardiology.

[6] Lee, K. Y. *et al*. CT Angiography Images of Coronary Artery Stenosis Provide a Better Prediction of Risk Than Traditional Risk Factors in Asymptomatic Individuals With Type 2 Diabetes: A Long-term Study of Clinical Outcomes. *Diabetes care*, 10.2337/dc16-1844 (2017). 10.2337/dc16-1844 28663384

[7] Nicholls SJ (2007). Coronary artery calcification and changes in atheroma burden in response to established medical therapies. Journal of the American College of Cardiology.

[8] Apfaltrer, G. *et al*. Impact on Image Quality and Radiation Dose of Third-Generation Dual-Source Computed Tomography of the Coronary Arteries. *The American journal of cardiology*, 10.1016/j.amjcard.2016.12.028 (2017). 10.1016/j.amjcard.2016.12.028 28233536

[9] Achenbach S (2010). Coronary computed tomography angiography with a consistent dose below 1 mSv using prospectively electrocardiogram-triggered high-pitch spiral acquisition. European heart journal.

[10] Alkadhi H (2010). Low-dose, 128-slice, dual-source CT coronary angiography: accuracy and radiation dose of the high-pitch and the step-and-shoot mode. Heart (British Cardiac Society).

[11] Stein PD, Yaekoub AY, Matta F, Sostman HD (2008). 64-slice CT for diagnosis of coronary artery disease: a systematic review. The American journal of medicine.

[12] Raggi P, Shaw LJ, Berman DS, Callister TQ (2004). Prognostic value of coronary artery calcium screening in subjects with and without diabetes. Journal of the American College of Cardiology.

[13] Shemesh J, Motro M, Morag-Koren N, Konen E, Grossman E (2012). Relation of coronary artery calcium to cardiovascular risk in patients with combined diabetes mellitus and systemic hypertension. The American journal of cardiology.

[14] Dedic A (2016). Prognostic Value of Coronary Computed Tomography Imaging in Patients at High Risk Without Symptoms of Coronary Artery Disease. The American journal of cardiology.

[15] Juutilainen A, Lehto S, Ronnemaa T, Pyorala K, Laakso M (2005). Type 2 diabetes as a “coronary heart disease equivalent”: an 18-year prospective population-based study in Finnish subjects. Diabetes care.

[16] Kannel WB, McGee DL (1979). Diabetes and cardiovascular disease. The Framingham study. Jama.

[17] Sarwar N (2010). Diabetes mellitus, fasting blood glucose concentration, and risk of vascular disease: a collaborative meta-analysis of 102 prospective studies. Lancet (London, England).

[18] Rivera JJ (2009). Association of traditional cardiovascular risk factors with coronary plaque sub-types assessed by 64-slice computed tomography angiography in a large cohort of asymptomatic subjects. Atherosclerosis.

[19] Adler AI (2000). Association of systolic blood pressure with macrovascular and microvascular complications of type 2 diabetes (UKPDS 36): prospective observational study. BMJ (Clinical research ed.).

[20] Rapsomaniki E (2014). Blood pressure and incidence of twelve cardiovascular diseases: lifetime risks, healthy life-years lost, and age-specific associations in 1.25 million people. Lancet (London, England).

[21] Davies MJ, Woolf N, Rowles PM, Pepper J (1988). Morphology of the endothelium over atherosclerotic plaques in human coronary arteries. British heart journal.

[22] Stary HC (1995). A definition of advanced types of atherosclerotic lesions and a histological classification of atherosclerosis. A report from the Committee on Vascular Lesions of the Council on Arteriosclerosis, American Heart Association. Arteriosclerosis, thrombosis, and vascular biology.

[23] Perk J (2012). European Guidelines on cardiovascular disease prevention in clinical practice (version 2012). The Fifth Joint Task Force of the European Society of Cardiology and Other Societies on Cardiovascular Disease Prevention in Clinical Practice (constituted by representatives of nine societies and by invited experts). European heart journal.

[24] Greenland P (2010). 2010 ACCF/AHA guideline for assessment of cardiovascular risk in asymptomatic adults: a report of the American College of Cardiology Foundation/American Heart Association Task Force on Practice Guidelines. Journal of the American College of Cardiology.

[25] Haberl R (2001). Correlation of coronary calcification and angiographically documented stenoses in patients with suspected coronary artery disease: results of 1,764 patients. Journal of the American College of Cardiology.

[26] Shaw LJ (2015). Long-Term Prognosis After Coronary Artery Calcification Testing in Asymptomatic Patients: A Cohort Study. Annals of internal medicine.

[27] Nakanishi R (2015). The relationship between coronary artery calcium score and the long-term mortality among patients with minimal or absent coronary artery risk factors. International journal of cardiology.

[28] Plank F (2014). The diagnostic and prognostic value of coronary CT angiography in asymptomatic high-risk patients: a cohort study. Open heart.

[29] Villines TC (2011). Prevalence and severity of coronary artery disease and adverse events among symptomatic patients with coronary artery calcification scores of zero undergoing coronary computed tomography angiography: results from the CONFIRM (Coronary CT Angiography Evaluation for Clinical Outcomes: An International Multicenter) registry. Journal of the American College of Cardiology.

[30] Schmermund A, Erbel R (2001). Unstable coronary plaque and its relation to coronary calcium. Circulation.

[31] Min JK (2010). Determinants of coronary calcium conversion among patients with a normal coronary calcium scan: what is the “warranty period” for remaining normal?. Journal of the American College of Cardiology.

[32] Groothuis JG (2012). Positive predictive value of computed tomography coronary angiography in clinical practice. International journal of cardiology.

[33] Arbab-Zadeh A (2012). Diagnostic accuracy of computed tomography coronary angiography according to pre-test probability of coronary artery disease and severity of coronary arterial calcification. The CORE-64 (Coronary Artery Evaluation Using 64-Row Multidetector Computed Tomography Angiography) International Multicenter Study. Journal of the American College of Cardiology.

[34] Centers for disease control and prevention, https://www.cdc.gov/nchs/nhis/tobacco/tobacco_glossary.htm. Accessed 26 September 2017.

[35] Rumberger JA, Brundage BH, Rader DJ, Kondos G (1999). Electron beam computed tomographic coronary calcium scanning: a review and guidelines for use in asymptomatic persons. Mayo Clinic proceedings.

[36] Agatston AS (1990). Quantification of coronary artery calcium using ultrafast computed tomography. Journal of the American College of Cardiology.

[37] Austen WG (1975). A reporting system on patients evaluated for coronary artery disease. Report of the Ad Hoc Committee for Grading of Coronary Artery Disease, Council on Cardiovascular Surgery, American Heart Association. Circulation.

[38] Gould KL, Lipscomb K, Hamilton GW (1974). Physiologic basis for assessing critical coronary stenosis. Instantaneous flow response and regional distribution during coronary hyperemia as measures of coronary flow reserve. The American journal of cardiology.

[39] Uren NG (1994). Relation between myocardial blood flow and the severity of coronary-artery stenosis. The New England journal of medicine.

[40] Kim WY (2001). Coronary magnetic resonance angiography for the detection of coronary stenoses. The New England journal of medicine.

[41] Leipsic J (2014). SCCT guidelines for the interpretation and reporting of coronary CT angiography: a report of the Society of Cardiovascular Computed Tomography Guidelines Committee. Journal of cardiovascular computed tomography.

